# A Note on the Connection Between Trek Rules and Separable Nonlinear Least Squares in Linear Structural Equation Models

**DOI:** 10.1007/s11336-022-09891-5

**Published:** 2022-12-25

**Authors:** Maximilian S. Ernst, Aaron Peikert, Andreas M. Brandmaier, Yves Rosseel

**Affiliations:** 1 Center for Lifespan Psychology, Max Planck Institute for Human Development, Lentzeallee 94, 14195 Berlin, Germany; 2 Center for Lifespan Psychology, Humboldt-Universität Zu Berlin, Berlin, Germany; 3 Max Planck UCL Centre for Computational Psychiatry and Ageing Research, Berlin, Germany and London, UK Lentzeallee 94, 14195 Berlin, Germany; 4 Department of Psychology, MSB Medical School Berlin, Berlin, Germany; 5 Department of Data Analysis, Ghent University, Ghent, Belgium

**Keywords:** Gaussian graphical model, graph theory, numerical optimization, least squares estimation, RAM notation

## Abstract

We show that separable nonlinear least squares (SNLLS) estimation is applicable to all linear structural equation models (SEMs) that can be specified in RAM notation. SNLLS is an estimation technique that has successfully been applied to a wide range of models, for example neural networks and dynamic systems, often leading to improvements in convergence and computation time. It is applicable to models of a special form, where a subset of parameters enters the objective linearly. Recently, Kreiberg et al. (Struct Equ Model Multidiscip J 28(5):725–739, 2021. 10.1080/10705511.2020.1835484) have shown that this is also the case for factor analysis models. We generalize this result to all linear SEMs. To that end, we show that undirected effects (variances and covariances) and mean parameters enter the objective linearly, and therefore, in the least squares estimation of structural equation models, only the directed effects have to be obtained iteratively. For model classes without unknown directed effects, SNLLS can be used to analytically compute least squares estimates. To provide deeper insight into the nature of this result, we employ trek rules that link graphical representations of structural equation models to their covariance parametrization. We further give an efficient expression for the gradient, which is crucial to make a fast implementation possible. Results from our simulation indicate that SNLLS leads to improved convergence rates and a reduced number of iterations.

**Supplementary Information:**

The online version contains supplementary material available at 10.1007/s11336-022-09891-5.

In the behavioral and social sciences, structural equation models (SEMs) have become widely accepted as a multivariate statistical tool for modeling the relation between latent and observed variables. Apart from maximum likelihood estimation, least squares (LS) estimation is a common approach for parameter estimation. In LS, parameters are estimated by minimizing a nonlinear function of the parameters and data. In practice, this problem is typically solved by applying generic nonlinear optimization techniques, such as Newton-type gradient descent approaches that iteratively minimize the objective function until convergence is reached. However, for some model classes, generic optimization algorithms can be adapted to make better use of the model structure and thus solve the problem more efficiently. For a particular type of models, the parameters *separate*, that is, one set of parameters enters the objective in a nonlinear way, while another set of parameters enters the objective linearly. For a vector of observations *y* and predictors *x* of size *m*, the objective is of the form

1 $$\begin{aligned} \sum _{i = 1}^m \left[ y_i - \sum _{j=1}^n \alpha _j \varphi _j(\beta , x_i)\right] ^2 \end{aligned}$$

where $\alpha \in \mathbb {R}^n, \beta \in \mathbb {R}^k$ are parameter vectors and the (nonlinear) functions $\varphi _j$ are continuously differentiable w.r.t. $\beta$. Golub and Pereyra (1973) showed that this kind of objective allows for a reformulation of the optimization problem, such that only the parameters $\beta$ have to be obtained iteratively, while the parameters $\alpha$ can be computed after the optimization in a single step. The procedure has been subsequently called separable nonlinear least squares (SNLLS). It has been successfully applied in many disciplines, and it has been observed that the reduced dimension of the parameter space can lead to reduced computation time, a reduced number of iterations and better convergence properties (Golub & Pereyra, 2003). Inspired by earlier work (Kreiberg et al., 2016, 2021) that showed that this procedure can also be applied to factor analysis models, we generalize their result to the entire class of linear structural equation models and give analytical gradients for the reduced optimization problem, which is central for an efficient implementation.

## Review of Concepts

In the following, we briefly review the notation for structural equation models, the generalized least squares estimator and the trek rules used to derive the model-implied covariance matrix.

### Linear Structural Equation Models

Linear structural equation models can be defined in RAM notation (reticular action model; McArdle & McDonald, 1984) as follows (we follow the notation from Drton, 2018): Let $x, \varepsilon$ be random vectors with values in $\mathbb {R}^m$ and

2 $$\begin{aligned} x = \varvec{\Lambda }x + \varepsilon \end{aligned}$$

where $\varvec{\Lambda }\in \mathbb {R}^{m \times m}$ is a matrix of constants or unknown (directed) parameters. Let $\varvec{\Omega }\in \mathbb {R}^{m \times m}$ be the covariance matrix of $\varepsilon$ and $\textbf{I}$ the identity matrix. If $\textbf{I}- \varvec{\Lambda }$ is invertible, Eq. 2 can be solved by $x = (\textbf{I}-\varvec{\Lambda })^{-1}\varepsilon$ with covariance matrix

3 $$\begin{aligned} \mathbb {V}[x] = \left( \textbf{I}-\varvec{\Lambda }\right) ^{-1} \varvec{\Omega }\left( \textbf{I}-\varvec{\Lambda }\right) ^{-T} \end{aligned}$$

If *x* is partitioned into a part $x_{\text {obs}}$ of $m_\textrm{obs}$ observed variables and $x_{\text {lat}}$ of $m_\textrm{lat}$ latent variables, we can reorder *x* such that $x = \left( x_{\text {obs}}^T \; x_{\text {lat}}^T\right) ^T$, and the covariance matrix of the observed variables is given by

4 $$\begin{aligned} \varvec{\Sigma }:=\mathbb {V}[x_{\text {obs}}] = \textbf{F}(\textbf{I}-\varvec{\Lambda })^{-1}\varvec{\Omega }(\textbf{I}-\varvec{\Lambda })^{-T}\textbf{F}^T\end{aligned}$$

where $\textbf{F}= \left[ {\textbf {I}}\,\big |\,{\textbf {0}}\right] \in \mathbb {R}^{m_\textrm{obs} \times (m_\textrm{obs} +m_\textrm{lat})}$ is a rectangular filter matrix. We denote the parameters by $\theta = \left( {\theta _{\Lambda }^{T} \Omega _{\Lambda }^{T}}\right) ^{T}\in \mathbb {R}^{q}$, partitioned into directed parameters from $\varvec{\Lambda }$ and undirected parameters from $\varvec{\Omega }$. (We call them directed or undirected parameters because they correspond to directed or undirected paths in the graph of the model.) If we want to stress that $\varvec{\Sigma }$ is a function of the parameters, we write $\varvec{\Sigma }(\theta )$. If we are also interested in the mean structure, we introduce a vector of (possibly zero) mean parameters $\gamma \in \mathbb {R}^m$ such that $x = \gamma + \varvec{\Lambda }x + \varepsilon$ and obtain

5 $$\begin{aligned} \mu :=\mathbb {E}[x_{obs}] = \textbf{F}(\textbf{I}- \varvec{\Lambda })^{-1}\gamma \end{aligned}$$

### Least Squares Estimation

The least squares objective function for $\theta$ is:

6 $$\begin{aligned} F_{\text {LS}} = (s - \sigma )^T \textbf{V}(s - \sigma ) \end{aligned}$$

where $\sigma = {{\,\textrm{vech}\,}}(\varvec{\Sigma })$ is the half-vectorization of $\varvec{\Sigma }$, that is, the vector of non-duplicated elements of the model-implied covariance matrix, $s = {{\,\textrm{vech}\,}}(\textbf{S})$ is the half-vectorization of the observed covariance matrix and $\textbf{V}$ is a fixed symmetric positive definite weight matrix. Specific forms of the weight matrix $\textbf{V}$ lead to commonly used special cases of this estimation technique: Generalized least squares estimation uses $\textbf{V}= \frac{1}{2} \, \textbf{D}^T\left( \textbf{S}^{-1} \otimes \textbf{S}^{-1}\right) \textbf{D}$ (where $\textbf{D}$ denotes the duplication matrix from Magnus and Neudecker (2019b)), asymptotic distribution-free estimation uses a consistent estimator of the asymptotic covariance matrix of *s*, and unweighted least squares estimation uses the identity matrix (Bollen, 1989; Browne, 1982, 1984).

### Trek Rules

To show that in SEM undirected effects enter the least squares objective linearly, we employ trek rules (Drton, 2018), which are path tracing rules used to derive the model-implied covariance between any pair of variables in a SEM (Boker et al., 2002). Various authors have proposed rules to link the graph to the covariance parametrization of the model. Here, we give the rules as put forward by Drton (2018), which are based on *treks* as basic building blocks (for an overview of alternative formulations see Mulaik, 2009). A trek $\tau$ from a node *i* to *j* is a path connecting them, where directed edges can be traveled forwards and backwards, but it is not allowed to walk from one arrowhead into another (without *colliding arrowheads*). A *top node* of a trek is a node which has only outgoing edges.

**Fig. 1:**
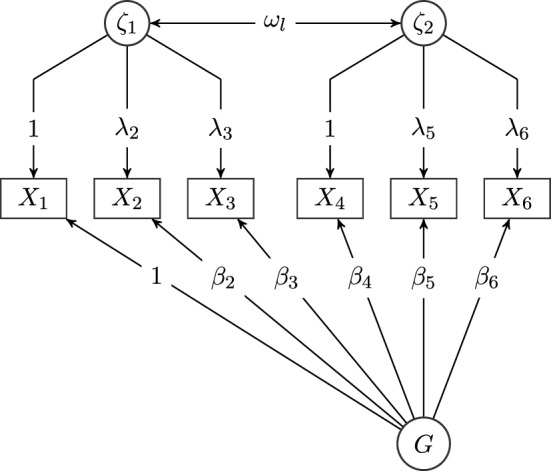
Graph of a bi-factor model with one general factor and two specific factors. Circles represent latent variables, and rectangles represent observed variables. Variances are omitted in this representation.

To derive an expression for the model-implied covariance between any two variables *i* and *j* based on the postulated SEM, we follow 4 steps:

1. Find all treks **from** *i* **to** *j*.
2. For each trek, multiply all parameters along it.
3. If a trek does not contain a covariance parameter, factor in the variance of the top node.
4. Add all obtained *trek monomials* from the different treks together.

Note that a trek is ordered in the sense that two treks containing exactly the same nodes and edges are considered different if they are traveled in a different order. In particular, each trek has a *source* (*i*) and a *target* (*j*), and a trek from *j* to *i* is considered to be a different trek, even if it contains exactly the same nodes and edges. Also note that variances are not considered to be edges in the mixed graph corresponding to the model (i.e., it is not allowed to travel variance edges). Therefore, all graphical representations of SEMs in this article omit variance edges, and it is required to factor them in according to rule 3 after the treks are collected.

#### Example

To illustrate how the model-implied covariances can be derived using trek rules, we give an example based on the graph shown in the path diagram in Fig. 1. To find the model-implied covariance between nodes $X_2$ and $X_6$ in the model shown in Fig. 1, we first find all treks from $X_2$ to $X_6$78We now compute the trek monomials for each trek. The second trek does not contain a covariance parameter, so we need to factor in the variance of the top node. We find the trek’s top node *G* and denote the variance parameter of G by $\omega _G$. Finally, we add the resulting trek monomials and we find that the model-implied covariance between $X_2$ and $X_6$ can be expressed as follows:

9 $$\begin{aligned} {{\,\textrm{cov}\,}}(X_2, X_6) = \lambda _2\omega _l\lambda _6 + \beta _2\omega _G\beta _6 \end{aligned}$$

As a second example, we derive the model-implied variance of $X_3$. Again, we first find all treks from $X_3$ to $X_3$:1011

12 $$\begin{aligned} X_3 \end{aligned}$$

All treks do not contain a covariance parameter, so we need to factor in the variance of the respective top nodes $\zeta _1$, *G* and $X_3$. We denote the variance parameters of $\zeta _1$ and $X_3$ by $\omega _{\zeta _1}$ and $\omega _3$ and add the resulting trek monomials to obtain

13 $$\begin{aligned} {{\,\textrm{var}\,}}(X_3) = \lambda _3^2\omega _{\zeta _1} + \beta _3^2\omega _G + \omega _3 \end{aligned}$$

#### Formal Definitions

We denote the elements of $\varvec{\Omega }$, the undirected effects between nodes *i* and *j*, by $\omega _{ij}$ and the elements of $\varvec{\Lambda }$, the directed effects, by $\lambda _{ij}$. Drton (2018) defines a *trek monomial* of a trek $\tau$ without a covariance parameter as

14 $$\begin{aligned} \tau (\varvec{\Lambda }, \varvec{\Omega }) = \omega _{i_0 i_0} \prod _{k \rightarrow l \in \tau } \lambda _{lk} \end{aligned}$$

where $i_0$ is the top node of the trek, and a trek monomial of a trek $\tau$ containing an undirected edge between $i_0$ and $j_0$ as

15 $$\begin{aligned} \tau (\varvec{\Lambda }, \varvec{\Omega }) = \omega _{i_0 j_0} \prod _{k \rightarrow l \in \tau } \lambda _{lk} \end{aligned}$$

(notice the swapped indices of $\lambda _{lk}$ compared to the formula in Drton because our $\varvec{\Lambda }$ corresponds to his $\varvec{\Lambda }^T$). With this, the elements of $\varvec{\Sigma }(\theta )$ are represented as a summation over treks. He proves that

16 $$\begin{aligned} \varvec{\Sigma }(\theta )_{ij} = \sum _{\tau \in {\mathcal {T}}(i,j)} \tau (\varvec{\Lambda }, \varvec{\Omega }) \end{aligned}$$

where ${\mathcal {T}}(i,j)$ is the set of all treks **from** *i* **to** *j*. It follows that the model-implied covariance is a sum of monomials of parameters. Because covariances between the error terms are not transitive, exactly one undirected parameter (variance or covariance) is present in each monomial. Therefore, if all the directed parameters were fixed, the model-implied covariance would be a linear function of the undirected parameters. This is what makes the SNLLS procedure applicable to structural equation models.

For later use, we also note that Drton gives the following expression:

17 $$\begin{aligned} (\textbf{I}- \varvec{\Lambda })^{-1}_{ij} = \sum _{\tau \in {\mathcal {P}}(j,i)} \prod _{k \rightarrow l \in \tau } \lambda _{lk} \end{aligned}$$

where ${\mathcal {P}}(j,i)$ is the set of directed paths from *j* to *i*. This is because we can write $(\textbf{I}- \varvec{\Lambda })^{-1} = \sum _{k = 0}^\infty \varvec{\Lambda }^k$, where the geometric series converges iff all eigenvalues of $\varvec{\Lambda }$ lie in $(-1, 1)$. (Further explanations about this and an excellent account of the connections between matrix algebra and graphs can be found in Kepner & Gilbert (2011).)

## Separable Nonlinear Least Squares for SEM

We first outline the proofs for the applicability of SNLLS to CFA as given by Golub and Pereyra (1973) and Kreiberg et al. (2021). Subsequently, we proof that SNLLS is applicable to linear structural equation models. We further extend the existing proofs to subsume models that contain a mean structure. Last, we derive analytic gradients that are central for efficient software implementations.

### Outline of Previous Work

To minimize Eq. 1, Golub and Pereyra (1973) define the matrix function

18 $$\begin{aligned} \Phi _{ij} :=\varphi _j(\beta , x_i) \end{aligned}$$

such that Eq. 1 can be written as

19 $$\begin{aligned} \Vert y - \Phi (\beta ) \alpha \Vert ^2 \end{aligned}$$

where $\Vert \cdot \Vert$ denotes the euclidean norm. For a fixed value of $\beta$, a solution for $\alpha$ can be obtained as $\alpha = \Phi ^+(\beta ) y$. They further proved that under the assumption that $\Phi (\beta )$ has constant rank near the solution, only the nonlinear parameters $\beta$ have to be obtained iteratively by replacing $\alpha$ and minimizing the modified objective

20 $$\begin{aligned} \left\Vert y - \Phi (\beta )\Phi ^+(\beta ) y \right\Vert ^2 \end{aligned}$$

where $\Phi ^+$ denotes the Moore–Penrose generalized inverse. Afterward, the least squares solution for the linear parameters $\alpha$ can be obtained as the standard least squares estimator ${{\,\mathrm{arg\,min}\,}}_{\alpha \in \mathbb {R}^n} \Vert \Phi (\hat{\beta })\alpha - y \Vert = \Phi ^+(\hat{\beta }) y$.

Kreiberg et al. (2021) showed that this procedure is applicable for CFA models (we reproduce their main results in our notation), as it is possible to rewrite the model-implied covariances $\sigma$ as a product of a matrix-valued function $\textbf{G}(\theta _{\varvec{\Lambda }})$ (that depends only on the directed parameters) and the undirected parameters $\theta _{\varvec{\Omega }}$, so the LS objective can be written as

21 $$\begin{aligned} F_{\text {LS}}&= (s - \sigma )^T \textbf{V}(s - \sigma ) \end{aligned}$$

22 $$\begin{aligned}&= \left( s - \textbf{G}(\theta _{\varvec{\Lambda }})\theta _{\varvec{\Omega }}\right) ^T \textbf{V}\left( s - \textbf{G}(\theta _{\varvec{\Lambda }})\theta _{\varvec{\Omega }} \right) \end{aligned}$$

23 $$\begin{aligned}&= \left\Vert s - \textbf{G}(\theta _{\varvec{\Lambda }})\theta _{\varvec{\Omega }}\right\Vert ^2_\textbf{V}\end{aligned}$$

They further stated that if $\theta _{\varvec{\Lambda }}$ is fixed, we know from standard linear least squares estimation that the minimizer for the undirected effects can be obtained as

24 $$\begin{aligned} \hat{\theta }_{\varvec{\Omega }} = \left( \textbf{G}( \theta _{\varvec{\Lambda }})^T \textbf{V}\textbf{G}( \theta _{\varvec{\Lambda }})\right) ^{-1} \textbf{G}( \theta _{\varvec{\Lambda }})^T\textbf{V}s \end{aligned}$$

Inserting Eq. 24 into Eq. 22 and simplifying, they obtained a new objective to be minimized:

25 $$\begin{aligned} \hat{\theta }_{\varvec{\Lambda }}&= \mathop {{{\,\mathrm{arg\,min}\,}}}\limits _{\theta _{\varvec{\Lambda }}} \left[ s^T\textbf{V}s - s^T\textbf{V}\textbf{G}(\theta _{\varvec{\Lambda }}) \left( \textbf{G}(\theta _{\varvec{\Lambda }})^T \textbf{V}\textbf{G}(\theta _{\varvec{\Lambda }})\right) ^{-1} \textbf{G}(\theta _{\varvec{\Lambda }})^T\textbf{V}s \right] \end{aligned}$$

26 $$\begin{aligned}&= \mathop {{{\,\mathrm{arg\,min}\,}}}\limits _{\theta _{\varvec{\Lambda }}} F_{\text {SNLLS}}(\theta _{\varvec{\Lambda }}) \end{aligned}$$

This objective only depends on the directed parameters $\theta _{\varvec{\Lambda }}$. After minimizing it to obtain a LS estimate $\hat{\theta }_{\varvec{\Lambda }}$, Eq. 24 can be used to obtain the LS estimate of $\theta _{\varvec{\Omega }}$. We would like to note they assume that $\textbf{G}$ has full rank, which is not a necessary assumption and can be relaxed using alternative formulations of Eqs. 24 and 25. To extend the method to general structural equation models, we have to derive $\textbf{G}(\theta _{\varvec{\Lambda }})$. We do that in the following for all models formulated in the RAM notation.

### Derivation of $\textbf{G}(\theta _{\varvec{\Lambda }})$

Since $\textbf{F}= \left[ {\textbf {I}}\,\big |\,{\textbf {0}}\right]$ with $\textbf{0} \in \mathbb {R}^{m_\textrm{obs} \times m_\textrm{lat}}$, the product $\textbf{F}\textbf{M}\textbf{F}^{T}$ for any $\textbf{M}\in \mathbb {R}^{m \times m}$ is equal to just deleting the last $m_\textrm{lat}$ rows and columns of $\textbf{M}$. We also note that for any matrices $\textbf{M}, \textbf{D}\in \mathbb {R}^{n \times n}$ we can write

27 $$\begin{aligned} \left( \textbf{M}\textbf{D}\textbf{M}^{T}\right) _{ij} = \sum _{k = 1}^n \sum _{l = 1}^n m_{il} \; d_{lk} \; m_{jk} \end{aligned}$$

With this in mind, we can rewrite the model-implied covariance matrix $\varvec{\Sigma }(\theta )$ as

28 $$\begin{aligned} \varvec{\Sigma }(\theta )_{ij}&= \bigg ( \textbf{F}{} & {} (\textbf{I}-\varvec{\Lambda })^{-1}{} & {} \varvec{\Omega }&(\textbf{I}-\varvec{\Lambda })^{-T}{} & {} \textbf{F}^T \bigg )_{ij} \end{aligned}$$

29 $$\begin{aligned}&= \bigg ({} & {} (\textbf{I}-\varvec{\Lambda })^{-1}{} & {} \varvec{\Omega }&(\textbf{I}-\varvec{\Lambda })^{-T}{} & {} \bigg )_{ij} \end{aligned}$$

30 $$\begin{aligned}&= \; \; \sum _{k = 1}^m \sum _{l = 1}^m{} & {} (\textbf{I}- \varvec{\Lambda })^{-1}_{il}{} & {} \omega _{lk}&(\textbf{I}- \varvec{\Lambda })^{-1}_{jk}{} & {} \end{aligned}$$

31 $$\begin{aligned}&= \; \; \sum _{k = 1}^m \sum _{l = 1}^m{} & {} \Big (\sum _{\tau \in \mathcal {P}(l,i)} \prod _{r \rightarrow s \in \tau } \lambda _{sr}\Big ){} & {} \omega _{lk}&\Big (\sum _{\tau \in \mathcal {P}(k,j)} \prod _{r \rightarrow s \in \tau } \lambda _{sr}\Big ){} & {} \end{aligned}$$

with $i,j \in \{1, \ldots , m_\textrm{obs}\}$. We now immediately see that each entry of $\varvec{\Sigma }$ is a sum of products of entries of $(\textbf{I}-\varvec{\Lambda })^{-1}$ and $\varvec{\Omega }$. More importantly, exactly one entry of $\varvec{\Omega }$ enters each term of the sum; if we keep all entries of $\varvec{\Lambda }$ fixed, each element in $\varvec{\Sigma }$ is a linear function of the entries of $\varvec{\Omega }$ and is therefore a linear function of the undirected parameters in $\varvec{\Omega }$ (under the assumption that $\varvec{\Omega }$ is linearly parameterized). As a result, the parameter vector $\theta$ is separable in two parts, $\theta _{\varvec{\Lambda }}$ from $\varvec{\Lambda }$ and $\theta _{\varvec{\Omega }}$ from $\varvec{\Omega }$, and $\theta _{\varvec{\Omega }}$ enters the computation of the model-implied covariance linearly. As stated before, this is the reason why we will be able to apply separable nonlinear least squares estimation to our problem. Before we proceed, we would like to introduce some notation. If ${\mathcal {F}}$ and ${\mathcal {G}}$ are tuples of length *n* and *m*, and *f* and *g* are functions, we define a column vector of length *n* as

32 $$\begin{aligned} \Bigg (\Big [f(i)\Big ]_{i \in {\mathcal {F}}}\Bigg ) = \begin{pmatrix} f({\mathcal {F}}_1) \\ f({\mathcal {F}}_2) \\ \ldots \\ f({\mathcal {F}}_n) \end{pmatrix} \end{aligned}$$

and a matrix of size $n \times m$ as

33 $$\begin{aligned} \Bigg (\Big [g(i,j)\Big ]_{i \in {\mathcal {F}},\; j \in {\mathcal {G}}}\Bigg ) = \begin{pmatrix} g({\mathcal {F}}_1, {\mathcal {G}}_1) &{}\quad g({\mathcal {F}}_1, {\mathcal {G}}_2) &{}\quad \ldots &{}\quad g({\mathcal {F}}_1, {\mathcal {G}}_m)\\ g({\mathcal {F}}_2, {\mathcal {G}}_1) &{}\quad g({\mathcal {F}}_2, {\mathcal {G}}_2) &{}\quad \ldots &{}\quad g({\mathcal {F}}_2, {\mathcal {G}}_m)\\ \ldots &{}\quad \ldots &{}\quad \ldots &{}\quad \ldots \\ g({\mathcal {F}}_n, {\mathcal {G}}_1) &{}\quad \ldots &{}\quad \ldots &{}\quad g({\mathcal {F}}_n, {\mathcal {G}}_m) \end{pmatrix} \end{aligned}$$

To make the subsequent steps easier to follow, we assume that there are no equality constraints between parameters in $\varvec{\Omega }$ and no constant terms in $\varvec{\Omega }$ different from 0. In Appendices A and B, we show how to lift those assumptions. We now further simplify Eq. 30: Since only nonzero entries of $\varvec{\Omega }$ (the parameters $\theta _{\varvec{\Omega }}$) contribute to the sum, we define ${\mathcal {C}}$ as the lower triangular indices of $\theta _{\varvec{\Omega }}$ in $\varvec{\Omega }$, i.e., ${\mathcal {C}}_i = (l, k) \in \mathbb {N}\times \mathbb {N}$ with $({\theta _{\varvec{\Omega }}})_i = \omega _{lk}$ and $l \ge k$. We now rewrite Eq. 30 by omitting all zero terms:

34 $$\begin{aligned} \varvec{\Sigma }(\theta )_{ij}&= \sum _{(l,k) \in {\mathcal {C}}} \left[ (\textbf{I}- \varvec{\Lambda })^{-1}_{il} \omega _{lk} (\textbf{I}- \varvec{\Lambda })^{-1}_{jk} + \delta _{k \ne l} \, (\textbf{I}- \varvec{\Lambda })^{-1}_{ik} \omega _{lk} (\textbf{I}- \varvec{\Lambda })^{-1}_{jl} \right] \end{aligned}$$

35 $$\begin{aligned}&= \left( \left[ (\textbf{I}- \varvec{\Lambda })^{-1}_{il} (\textbf{I}- \varvec{\Lambda })^{-1}_{jk} + \delta _{k \ne l} \, (\textbf{I}- \varvec{\Lambda })^{-1}_{ik} (\textbf{I}- \varvec{\Lambda })^{-1}_{jl}\right] _{(l,k) \in {\mathcal {C}}} \right) ^T \; \theta _{\varvec{\Omega }} \end{aligned}$$

where $\delta _{k \ne l}$ is an indicator function that takes the value 1 if $k \ne l$ and 0 otherwise. Since we are only interested in the non-duplicated elements $\sigma$ of $\varvec{\Sigma }$, we define another index tuple ${\mathcal {D}}$ that denotes the indices of the original position of $\sigma _k$ in $\varvec{\Sigma }$, i.e., ${\mathcal {D}}_k = (i, j)$ such that $\sigma _k = \varvec{\Sigma }_{ij}$. This allows us to stack the expression we just found for $\varvec{\Sigma }_{ij}$ rowwise to get

36 $$\begin{aligned} \sigma&= \left( \Big [\varvec{\Sigma }_{ij} \Big ]_{(i, j) \in {\mathcal {D}}} \right) \end{aligned}$$

37 $$\begin{aligned}&= \left( \left[ (\textbf{I}- \varvec{\Lambda })^{-1}_{il} (\textbf{I}- \varvec{\Lambda })^{-1}_{jk} + \delta _{k \ne l} \, (\textbf{I}- \varvec{\Lambda })^{-1}_{ik} (\textbf{I}- \varvec{\Lambda })^{-1}_{jl}\right] _{(i, j) \in {\mathcal {D}}, \; (l,k) \in {\mathcal {C}}} \; \right) \theta _{\varvec{\Omega }} \end{aligned}$$

38 $$\begin{aligned}&= \textbf{G}(\theta _{\varvec{\Lambda }}) \; \theta _{\varvec{\Omega }} \end{aligned}$$

where $\textbf{G}(\theta _{\varvec{\Lambda }}) \in \mathbb {R}^{\dim (\sigma ) \times \dim (\theta _{\varvec{\Omega }})}$. (We let $\dim (\cdot )$ of a vector denote its number of elements, i.e., the dimension of the underlying (finite-dimensional) vector space.)

Even though this expression may appear involved, it is in fact easy to compute. Before the optimization procedure starts, we store ${\mathcal {C}}$ by looking up the positions of the parameters in $\varvec{\Omega }$ and also store ${\mathcal {D}}$. At each step of the optimization procedure, to compute $\textbf{G}(\theta _{\varvec{\Lambda }})$, we now compute $(\textbf{I}- \varvec{\Lambda })^{-1}$ first and then loop through the entries ${\mathcal {C}}$ and ${\mathcal {D}}$ to compute each entry of $\textbf{G}(\theta _{\varvec{\Lambda }})$ according to Eq. 37. We note that $\textbf{G}$ will typically be sparse; therefore, it is advisable to analyze its sparsity pattern previous to the optimization, and only loop through nonzero values.

In Appendix D, we present a different way of obtaining $\textbf{G}(\theta _{\varvec{\Lambda }})$ and the gradients, which mimics the approach of Kreiberg et al. (2021). However, the expressions obtained here are computationally more efficient, as the ones in the appendix contain very large Kronecker products.

### Mean Structures

If the model contains mean parameters, we partition the parameter vector $\theta$ into three parts: $\theta _{\varvec{\Lambda }}$ and $\theta _{\varvec{\Omega }}$ as before, and $\theta _\gamma$ from the mean vector $\gamma$. From Eq. 5, we directly see that the model-implied mean vector $\mu (\theta )$ is a linear function of $\theta _\gamma$. If we let ${\mathcal {A}}$ denote the indices of the parameters $\theta _{\gamma }$ in $\gamma$, i.e., for $i = {\mathcal {A}}_{j}$ we have $({\theta _{\gamma }})_j$ = $\gamma _i$, we obtain the formula

39 $$\begin{aligned} \mu = \left( \left[ (\textbf{I}- \varvec{\Lambda })^{-1}_{ij}\right] _{i \in (1, \ldots , m_\textrm{obs}), \; j \in {\mathcal {A}}} \right) \; \theta _{\gamma }\end{aligned}$$

We now make a slight change in notation: For the previously obtained $\textbf{G}(\theta )$-matrix, we write $\textbf{G}_\sigma$ instead and define $\textbf{G}_\mu :=\left( \left[ (\textbf{I}- \varvec{\Lambda })^{-1}_{ij}\right] _{i \in (1, \ldots , m_\textrm{obs}), \; j \in {\mathcal {A}}} \right)$. Using a formulation of the least squares objective that also includes a mean structure, we see that40with

41 $$\begin{aligned} \textbf{G}:=\left[ \begin{array}{cc} \textbf{G}_\sigma &{}\quad \textbf{0} \\ \textbf{0} &{}\quad \textbf{G}_\mu \end{array}\right] \end{aligned}$$

It follows that in addition to the undirected parameters, the mean parameters also do not have to be optimized iteratively but can instead be computed analytically after the iterative optimization is completed.

### Gradient of the SNLLS Objective

There are computationally efficient expression to compute the SNLLS objective and its gradient analytically (Kaufman, 1975; O’Leary & Rust, 2013). Because numerical approximations of the gradient are often slow and may become numerically unstable, we derive an analytical expression for the part of the gradient that is specific to SEMs. We use the notation and methods from Magnus and Neudecker (2019a) and denote the differential by ${{\,\mathrm{\textsf{d}}\,}}$ and the Jacobian by ${{\,\mathrm{\textsf{D}}\,}}$. The Jacobian of a matrix function $\textbf{M}$ with respect to a vector *x* is defined as ${{\,\mathrm{\textsf{D}}\,}}\textbf{M}= \frac{\partial {{\,\textrm{vec}\,}}\textbf{M}}{\partial x^{T}}$. In the approaches by Kaufman (1975) and O’Leary and Rust (2013), the gradient of the SNLSS objective is expressed in terms of the partial derivatives of the entries of $\textbf{G}$ w.r.t the nonlinear parameters, i.e., ${{\,\mathrm{\textsf{D}}\,}}\textbf{G}$. In order to be able to implement such efficient approaches in practice, we derive ${{\,\mathrm{\textsf{D}}\,}}\textbf{G}$ here. We also give the full gradient of Eq. 25 for completeness in Appendix C, although in practice, a more efficient expression from the cited literature can be used (which also does not assume $\textbf{G}$ to have full rank). For reasons of clarity, we here only consider the case without mean structure, e.g., $\textbf{G}= \textbf{G}_\sigma$. This is because the derivative of $\textbf{G}_\mu$ is similar to obtain and we do not want to make the derivation unnecessarily technical.

Let ${\mathcal {E}}$ denote the indices of $\theta _{\varvec{\Lambda }}$ in $\varvec{\Lambda }$, i.e., ${\mathcal {E}}_k = (i,j)$ such that $\varvec{\Lambda }_{ij} = ({\theta _{\varvec{\Lambda }}})_k$. We note that

42 $$\begin{aligned} \frac{\partial (\textbf{I}- \varvec{\Lambda })^{-1}_{kl}}{\partial \varvec{\Lambda }_{ij}} = (\textbf{I}- \varvec{\Lambda })^{-1}_{ki}(\textbf{I}- \varvec{\Lambda })^{-1}_{jl} \end{aligned}$$

With this, we derive the partial derivatives of each entry of $\textbf{G}$ in terms of the matrix $(\textbf{I}- \varvec{\Lambda })^{-1}$ as

43 $$\begin{aligned} \frac{\partial \textbf{G}_{r, s}}{\partial ({\theta _{\varvec{\Lambda }}})_n}&= \frac{\partial }{\partial ({\theta _{\varvec{\Lambda }}})_n} \left[ (\textbf{I}- \varvec{\Lambda })^{-1}_{il} (\textbf{I}- \varvec{\Lambda })^{-1}_{jk} +\delta _{k \ne l} \, (\textbf{I}- \varvec{\Lambda })^{-1}_{ik} (\textbf{I}- \varvec{\Lambda })^{-1}_{jl}\right] \end{aligned}$$

44 $$\begin{aligned}&= \left[ \frac{\partial }{\partial ({\theta _{\varvec{\Lambda }}})_n}(\textbf{I}- \varvec{\Lambda })^{-1}_{il} (\textbf{I}- \varvec{\Lambda })^{-1}_{jk} + (\textbf{I}- \varvec{\Lambda })^{-1}_{il} \frac{\partial }{\partial ({\theta _{\varvec{\Lambda }}})_n}(\textbf{I}- \varvec{\Lambda })^{-1}_{jk}\right] \nonumber \\&\quad +\delta _{k \ne l} \left[ \frac{\partial }{\partial ({\theta _{\varvec{\Lambda }}})_n}(\textbf{I}- \varvec{\Lambda })^{-1}_{ik} (\textbf{I}- \varvec{\Lambda })^{-1}_{jl} + (\textbf{I}- \varvec{\Lambda })^{-1}_{ik} \frac{\partial }{\partial ({\theta _{\varvec{\Lambda }}})_n}(\textbf{I}- \varvec{\Lambda })^{-1}_{jl} \right] \end{aligned}$$

45 $$\begin{aligned}&= \left[ (\textbf{I}- \varvec{\Lambda })^{-1}_{iu} (\textbf{I}- \varvec{\Lambda })^{-1}_{vl} (\textbf{I}- \varvec{\Lambda })^{-1}_{jk} + (\textbf{I}- \varvec{\Lambda })^{-1}_{il} (\textbf{I}- \varvec{\Lambda })^{-1}_{ju} (\textbf{I}- \varvec{\Lambda })^{-1}_{vk} \right] \nonumber \\&\quad +\delta _{k \ne l} \left[ (\textbf{I}- \varvec{\Lambda })^{-1}_{iu} (\textbf{I}- \varvec{\Lambda })^{-1}_{vk} (\textbf{I}- \varvec{\Lambda })^{-1}_{jl} + (\textbf{I}- \varvec{\Lambda })^{-1}_{ik} (\textbf{I}- \varvec{\Lambda })^{-1}_{ju} (\textbf{I}- \varvec{\Lambda })^{-1}_{vl} \right] \end{aligned}$$

with $(i, j) = {\mathcal {D}}_r$, $(l, k) = {\mathcal {C}}_s$, and $(u, v) = {\mathcal {E}}_n$. Since $\textbf{G}$ is of dimension $\dim (\sigma ) \times \dim (\theta _{\varvec{\Omega }})$, with $k = \dim (\sigma )$ we have

46 $$\begin{aligned} {{\,\textrm{vec}\,}}(\textbf{G})_t = \textbf{G}_{t - k\lfloor (t-1)/k) \rfloor , \; \lceil t / k \rceil } \end{aligned}$$

and we obtain ${{\,\mathrm{\textsf{D}}\,}}\textbf{G}\in \mathbb {R}^{\dim (\sigma )\dim (\theta _{\varvec{\Omega }}) \times \dim (\theta _{\varvec{\Lambda }})}$ as

47 $$\begin{aligned} {{\,\mathrm{\textsf{D}}\,}}\textbf{G}= \frac{\partial {{\,\textrm{vec}\,}}\textbf{G}}{\partial \theta _{\varvec{\Lambda }}^T} = \left( \left[ \frac{\partial \textbf{G}_{t - k\lfloor (t-1)/k) \rfloor , \; \lceil t / k \rceil }}{\partial ({\theta _{\varvec{\Lambda }}})_n}\right] _{t \in (1, \ldots , \dim (\sigma )\dim (\theta _{\varvec{\Omega }})), \; n \in (1, \ldots , \dim (\theta _{\varvec{\Lambda }}))}\right) \end{aligned}$$

To facilitate software implementation, we give a way to compute ${{\,\mathrm{\textsf{D}}\,}}\textbf{G}$ in pseudocode in Algorithm 1. In practice, ${{\,\mathrm{\textsf{D}}\,}}\textbf{G}$ will typically contain many zero values. Therefore, it is advisable to analyze the sparsity pattern of ${{\,\mathrm{\textsf{D}}\,}}\textbf{G}$ before the optimization procedure begins and to only compute the nonzero values of ${{\,\mathrm{\textsf{D}}\,}}\textbf{G}$ at each iteration. Also note that the entries of ${{\,\mathrm{\textsf{D}}\,}}\textbf{G}$ are continuous w.r.t $\theta _{\varvec{\Lambda }}$, since they are sums of products of entries of the inverse $(\textbf{I}- \varvec{\Lambda })^{-1}$, which is continuous.
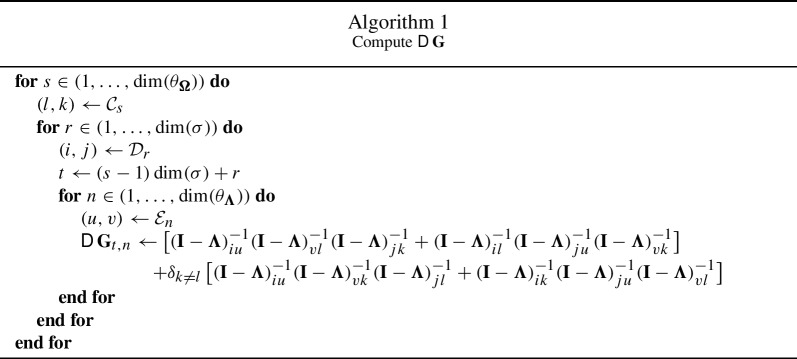


## Discussion

We have shown that separable nonlinear least squares is applicable to generalized least squares estimation of structural equation models formulated in the RAM notation. We have also shown a connection to path tracing rules in the form of trek rules. Note that when the same weight matrix is used, the point estimates obtained by SNLLS and LS are identical. Therefore, standard errors and test statistics are obtained using the same methods available for regular least squares estimation. In the following, we would like to discuss the two major benefits of using SNLLS for SEM: better convergence properties and a reduction in the computation time for parameter estimation.

### Convergence

An important issue in SEM is convergence problems, especially in small samples (De Jonckere & Rosseel, 2022). If the optimizer fails to converge, no parameter estimates can be obtained. Using the SNLLS objective should lead to fewer convergence problems than LS, since only the directed parameters need to be estimated iteratively. Therefore, only the subset of directed parameters requires starting values. In many models, most of the directed parameters are factor loadings, and we can obtain very good starting values for them with the FABIN 3 estimator (Hägglund, 1982). Also, Ruhe and Wedin (1980) and Golub and Pereyra (2003) give additional proofs and reasons for why the reduced optimization problem of SNLLS should in principle be better behaved than the full LS problem. Additionally, for the class of models without unknown directed parameters, convergence problems should be eliminated altogether, as the estimator of the mean and (co)variance parameters can be computed analytically. Most prominently, this features many types of latent growth curve models.

**Fig. 2:**
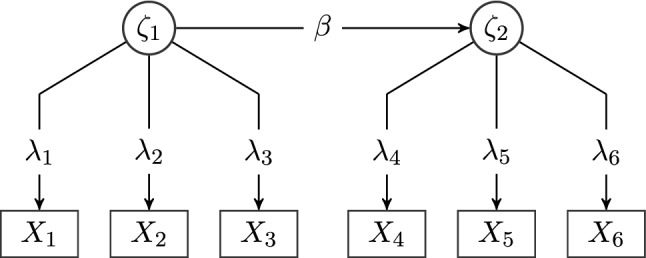
The structural equation model used to compare convergence properties of SNLLS and GLS estimation, with two latent variables, $\zeta _{1}$ and $\zeta _{2}$. Variances are omitted in this representation. The population values are the same as in De Jonckere and Rosseel (2022): $\lambda _{1} = \lambda _{4} = 1$, $\lambda _{2} = \lambda _{5} = 0.8$, $\lambda _{3} = \lambda _{6} = 0.6$, $\beta =0.25$, and all error variances are set to 1

**Fig. 3:**
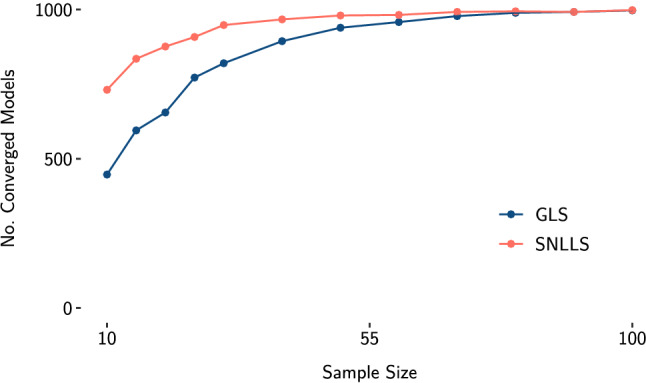
Simulation results—number of converged replications out of 1000. GLS, generalized least squares; SNLLS, separable nonlinear least squares

**Fig. 4:**
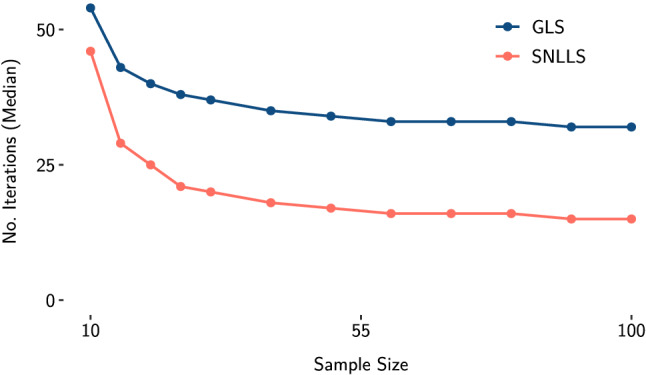
Simulation results—median number of iterations by sample size. GLS, generalized least squares; SNLLS, separable nonlinear least squares

To investigate the convergence properties of SNLLS in SEM, we ran a small simulation. We used the model in Fig. 2 to draw 1000 random data sets for varying sample sizes (*N* = 10 to *N* = 100) under the assumption of multivariate normality with zero expectation and the model-implied covariance induced by the parameters. The sample size and the factor loadings are deliberately chosen to be small to achieve a setting where non-convergence often occurs. We fitted the true model to each sample with generalized least squares (GLS; Bollen, 1989) and SNLLS estimation. All analyses were done in the programming language R (R Core Team, 2021). For GLS estimation, we used lavaan (Rosseel, 2012). The plots were created with ggplot2 (Wickham, 2016), and the data were prepared with dplyr (Wickham et al., 2021). In Fig. 3 we report the number of converged models for each sample size. In Fig. 4, we report the median number of iterations needed until convergence for each sample size. Using SNLLS effectively halved the median number of iterations until convergence for most sample sizes and more than halved the number of non-converged models for most sample sizes. This indicates that SNLLS might be a useful alternative for applied researchers to consider if they encounter convergence problems.

### Computation Time

The benefits of SNLLS estimation, specifically the reduced dimensionality of the parameter space, better starting values and fewer iterations to convergence, could lead to reduced computation times. However, the computation of the SNLLS objective function and gradient is also more costly, so the cost per iteration can be higher. In sum, the question whether SNLLS estimation is faster in actual time spent in the optimization hinges upon several aspects, such as the actual implementation of the gradient, meta-parameters of the optimizer and model complexity.

Kreiberg et al. (2021) stated that estimation by SNLLS will typically be multiple times faster than LS when the reduced parameter space is much smaller than the original one. They conducted a simulation study, where they fitted a number of CFA models and concluded that the estimation time is bigger for LS than for SNLLS as the number of estimated parameters increases. Even though their simulation is useful to illustrate the potential benefits of SNLLS, it seems unfit to us to make a case for a general reduction in computation time when using SNLLS in modern software. The gradient computation in the simulation was based on a finite difference approximation in both the LS and the SNLLS condition. In existing software (Rosseel, 2012; von Oertzen et al., 2015), analytic gradients are implemented for LS estimation, so the authors compare against a straw man that would not be used in practice if computational efficiency is important. In addition, centered finite differences takes 2*q* calls to the objective function per computation of the gradient, where *q* is the number of parameters. Since SNLLS results in a smaller parameter space, their method of differentiation favors the SNLLS procedure.

It remains to implement a competitive version of SNLLS optimization for SEM using the analytic gradients derived in this paper to be able to do a realistic simulation to investigate whether SNLLS outperforms the LS estimator in practice. However, there is a large body of research concerning the efficient implementation of SNLLS (see, for example, Kaufman, 1975; O’Leary and Rust, 2013); writing competitive software for SNLLS in SEMs would be a research topic on its own. Therefore, we only give simulation results concerning the improvement of convergence rates and the number of iterations in this paper. As noted previously, for the class of models without unknown directed parameters, the estimator of the mean and (co)variance parameters can be computed in a single step. As a result, those models should especially benefit from lower computation times.

### An Outlook on Maximum Likelihood Estimation

**Fig. 5:**
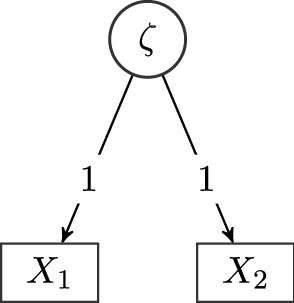
Graph of a simplistic example model with one latent variable, measured by two indicators. The model contains no unknown directed effects and only two observed variables to allow for an easily traceable computation of the inverse of the model-implied covariance matrix. All variances are treated as unknown parameters

If the assumption of multivariate normality is tenable, another method of obtaining parameter estimates is maximum likelihood estimation. Here, we briefly discuss to what extent our results may have an impact on maximum likelihood optimization of SEMs. In least squares estimation with a fixed weight matrix, we saw that the undirected parameters $\theta _{\varvec{\Omega }}$ and the mean parameters $\theta _\gamma$ enter the objective linearly. For maximum likelihood estimation, we believe it is not possible to factor out the undirected parameters (for most models used in practice). This is because the likelihood of the normal distribution

48 $$\begin{aligned} \phi (x) = \left( (2\pi )^{m_\textrm{obs}} \det \varvec{\Sigma }\right) ^{-\frac{1}{2}} \exp \left( -\frac{1}{2} (x - \mu )^T \varvec{\Sigma }^{-1} (x - \mu ) \right) \end{aligned}$$

depends on the inverse of the model-implied covariance matrix. For the simplistic example model depicted in Fig. 5, we derive the model-implied covariance matrix as

49 $$\begin{aligned} \varvec{\Sigma }= \begin{pmatrix} \omega _l + \omega _1 &{} \omega _l\\ \omega _l &{} \omega _l + \omega _2 \end{pmatrix} \end{aligned}$$

and the inverse can be computed as

50 $$\begin{aligned} \varvec{\Sigma }^{-1} = (\det \varvec{\Sigma })^{-1} {{\,\textrm{adj}\,}}\varvec{\Sigma }\end{aligned}$$

where ${{\,\textrm{adj}\,}}$ refers to the adjugate matrix, so in our example,

51 $$\begin{aligned} \det \varvec{\Sigma }= (\omega _l + \omega _1)(\omega _l + \omega _2) - \omega _l^2 = \omega _1\omega _l + \omega _2\omega _l + \omega _1\omega _2 \end{aligned}$$

and

52 $$\begin{aligned} \varvec{\Sigma }^{-1} = (\omega _1\omega _l + \omega _2\omega _l + \omega _1\omega _2)^{-1} \begin{pmatrix} \omega _l + \omega _2 &{}\quad -\omega _l\\ -\omega _l &{}\quad \omega _l + \omega _1 \end{pmatrix} \end{aligned}$$

We see that $\theta _{\varvec{\Omega }}$ enters the determinant and therefore the inverse of $\varvec{\Sigma }$ in a nonlinear way. In general, the Leibniz Formula for the determinant gives

53 $$\begin{aligned} \det \varvec{\Sigma }= \sum _{\pi \in {\mathcal {S}}_{m_\textrm{obs}}} {{\,\textrm{sgn}\,}}(\pi ) \prod _{i = 1}^{m_\textrm{obs}} \varvec{\Sigma }_{i,\pi (i)} \end{aligned}$$

where ${\mathcal {S}}_{m_\textrm{obs}}$ denotes the symmetric group. Since this formula multiplies entries of $\varvec{\Sigma }$, and we saw in Eq. 30 that the entries of $\varvec{\Sigma }$ depend on the undirected parameters, it is very likely that those form a product and enter the objective in a nonlinear way. However, for the mean parameters, the picture may be different and we leave this for future work. If the model is saturated (e.g., has zero degrees of freedom), the least squares estimates are the same as the maximum likelihood estimates, since $\textbf{S} = \varvec{\Sigma }(\hat{\theta }_\textrm{ML}) = \varvec{\Sigma }(\hat{\theta }_\textrm{LS})$. Also, Lee and Jennrich (1979) showed that maximum likelihood estimation can be obtained as a form of iteratively reweighted least squares if $\textbf{V}$ is a function of the parameters:

54 $$\begin{aligned} \textbf{V}= \frac{1}{2}\textbf{D}^T\left( \varvec{\Sigma }^{-1} \otimes \varvec{\Sigma }^{-1}\right) \textbf{D}\end{aligned}$$

where $\textbf{D}$ denotes the duplication matrix from Magnus and Neudecker (2019b). Another way of obtaining ML estimates with SNLLS would therefore be to minimize the SNLLS objective and use the obtained $\varvec{\Sigma }$ to update the weight matrix $\textbf{V}$ as given in Eq. 54. SNLLS could then be rerun with the updated weight matrix, and the weight matrix be updated again, until $\varvec{\Sigma }$ converges to $\varvec{\Sigma }(\hat{\theta }_\textrm{ML})$. However, we would like to note that this procedure is probably computationally quite inefficient.

### Conclusion

We generalized separable nonlinear least squares estimation to all linear structural equation models that can be specified in the RAM notation, particularly those including a mean structure. We explained this result with the help of trek rules and the non-transitivity of the covariances of the error terms, providing deeper insight into the algebraic relations between the parameters of SEMs. We further derived analytic gradients and explained why they are of central importance to obtain a competitive implementation. Our simulation indicates that SNLLS leads to improvements in convergence rate and number of iterations. It remains for future research to investigate the computational costs empirically. We also showed why it is unlikely that undirected parameters enter the maximum likelihood objective linearly. Thus, another line of research could be concerned with the applicability of SNLLS to the mean parameters in maximum likelihood estimation and the relationship of SNLLS to other decomposition methods for maximum likelihood estimation (Pritikin et al., 2017, 2018). Further research might also examine whether SNLLS is applicable to multilevel models. SNLLS promises better convergence rates for least squares parameter estimation in SEM and, with an efficient implementation, also reduced computation times. This result is important in its own right but may as well serve as a first step for generating starting values for subsequent ML estimation.

### Supplementary Information

Below is the link to the electronic supplementary material.

Supplementary file 1 (txt 3 KB)

Supplementary file 2 (txt 4 KB)

## Appendix A: Equality Constraints

Kreiberg et al. (2021) showed how to incorporate equality constraints in CFA models. Because their proof follows a different approach, we show how to incorporate equality constraints in our expressions. Since the SNLLS objective only depends on $\theta _{\varvec{\Lambda }}$, constraints in $\theta _{\varvec{\Omega }}$ and $\theta _\gamma$ can be difficult to implement. However, simple equality constraints (e.g., $\theta _j = \theta _i$) are feasible under SNLLS. Since $\sigma = \textbf{G}\, \theta _{\varvec{\Omega }, \gamma }$, we see that if two (or more) parameters in $\theta _{\varvec{\Omega }, \gamma }$ are equal, we can delete all but one occurrence from the parameter vector and add the relevant columns in $\textbf{G}$ together, e.g.,

A1 $$\begin{aligned} \begin{pmatrix} a&b&c \end{pmatrix} \begin{pmatrix} d \\ e \\ d \end{pmatrix} = ad + be + cd = (a+c)d + be = \begin{pmatrix} a + c&b \end{pmatrix} \begin{pmatrix} d \\ e \end{pmatrix} \end{aligned}$$

Or, put differently, if we allow the index tuples ${\mathcal {C}}$ and ${\mathcal {A}}$ to have sets of indices as entries, i.e., ${\mathcal {C}}_i = \{(k, l) \in \mathbb {N}\times \mathbb {N}\; | \; \theta _{\varvec{\Omega }_i} = \omega _{kl} \wedge k \ge l\}$, we obtain

A2 $$\begin{aligned} \textbf{G}_\sigma = \left( \left[ \sum _{(l,k) \in c} (\textbf{I}- \varvec{\Lambda })^{-1}_{il} (\textbf{I}- \varvec{\Lambda })^{-1}_{jk} + \delta _{k \ne l} \, (\textbf{I}- \varvec{\Lambda })^{-1}_{ik} (\textbf{I}- \varvec{\Lambda })^{-1}_{jl}\right] _{(i, j) \in {\mathcal {D}}, \; c \in {\mathcal {C}}} \right) \end{aligned}$$

An expression for $\textbf{G}_\mu$ can be obtained in a similar way.

## Appendix B: Constants in $\varvec{\Omega }$

To handle constants in $\varvec{\Omega }$ different from zero, we introduce *c* as the vector of constant nonzero entries in $\varvec{\Omega }$ and ${\mathcal {E}}$ as the lower triangular indices of *c* in $\varvec{\Omega }$. Further, define

B1 $$\begin{aligned} \textbf{G}_c :=\left( \left[ (\textbf{I}- \varvec{\Lambda })^{-1}_{il} (\textbf{I}- \varvec{\Lambda })^{-1}_{jk} + \delta _{k \ne l} \, (\textbf{I}- \varvec{\Lambda })^{-1}_{ik} (\textbf{I}- \varvec{\Lambda })^{-1}_{jl}\right] _{(i, j) \in {\mathcal {D}}, \; (l,k) \in {\mathcal {E}}} \; \right) \end{aligned}$$

with ${\mathcal {D}}$ defined as in Eq. 36, i.e., the indices of the original position of $\sigma _k$ in $\varvec{\Sigma }$. This allows us to modify Eq. 38 to

B2 $$\begin{aligned} \sigma = \textbf{G}(\theta _{\varvec{\Lambda }}) \; \theta _{\varvec{\Omega }} \; + \; \textbf{G}_c \; c \end{aligned}$$

and reformulate the least squares objective as

B3 $$\begin{aligned} F_{\text {LS}}&= (s - \sigma )^T \textbf{V}(s - \sigma ) \end{aligned}$$

B4 $$\begin{aligned}&= \left( s - \left( \textbf{G}(\theta _{\varvec{\Lambda }})\theta _{\varvec{\Omega }} + \textbf{G}_c c \right) \right) ^T \textbf{V}\left( s - \left( \textbf{G}(\theta _{\varvec{\Lambda }})\theta _{\varvec{\Omega }} + \textbf{G}_c c\right) \right) \end{aligned}$$

B5 $$\begin{aligned}&= \left( \left( s - \textbf{G}_c c \right) - \textbf{G}(\theta _{\varvec{\Lambda }})\theta _{\varvec{\Omega }} \right) ^T \textbf{V}\left( \left( s - \textbf{G}_c c \right) - \textbf{G}(\theta _{\varvec{\Lambda }})\theta _{\varvec{\Omega }}\right) \end{aligned}$$

B6 $$\begin{aligned}&= \left\Vert s' - \textbf{G}(\theta _{\varvec{\Lambda }})\theta _{\varvec{\Omega }}\right\Vert ^2_\textbf{V}\end{aligned}$$

with $s' = \left( s - \textbf{G}_c c \right)$. For a fixed value of $\theta _{\varvec{\Lambda }}$, we can now again solve for $\theta _{\varvec{\Omega }}$.

## Appendix C: Gradient of the SNLLS Objective

Let $a^T:= s^T\textbf{V}\textbf{G}\left( \textbf{G}^T \textbf{V}\textbf{G}\right) ^{-1}$. We derive the differential as

C1 $$\begin{aligned} {{\,\mathrm{\textsf{d}}\,}}F_\text {SNLLS}&= {{\,\mathrm{\textsf{d}}\,}}\left( s^T\textbf{V}s - s^T\textbf{V}\textbf{G}\left( \textbf{G}^T\textbf{V}\textbf{G}\right) ^{-1}\textbf{G}^T\textbf{V}s \right) \nonumber \\&= - {{\,\mathrm{\textsf{d}}\,}}\left( s^T\textbf{V}\textbf{G}\left( \textbf{G}^T\textbf{V}\textbf{G}\right) ^{-1}\textbf{G}^T\textbf{V}s \right) \nonumber \\&= - s^T\textbf{V}{{\,\mathrm{\textsf{d}}\,}}\textbf{G}\left( \textbf{G}^T\textbf{V}\textbf{G}\right) ^{-1}\textbf{G}^T\textbf{V}s \nonumber \\&\quad - s^T\textbf{V}\textbf{G}{{\,\mathrm{\textsf{d}}\,}}\left( \textbf{G}^T\textbf{V}\textbf{G}\right) ^{-1}\textbf{G}^T\textbf{V}s \nonumber \\&\quad - s^T\textbf{V}\textbf{G}\left( \textbf{G}^T\textbf{V}\textbf{G}\right) ^{-1} {{\,\mathrm{\textsf{d}}\,}}\textbf{G}^T\textbf{V}s \nonumber \\&= -2 \; s^T\textbf{V}{{\,\mathrm{\textsf{d}}\,}}\textbf{G}\left( \textbf{G}^T\textbf{V}\textbf{G}\right) ^{-1}\textbf{G}^T\textbf{V}s \nonumber \\&\quad + s^T\textbf{V}\textbf{G}\left( \textbf{G}^T\textbf{V}\textbf{G}\right) ^{-1} {{\,\mathrm{\textsf{d}}\,}}\left( \textbf{G}^T\textbf{V}\textbf{G}\right) \left( \textbf{G}^T\textbf{V}\textbf{G}\right) ^{-1}\textbf{G}^T\textbf{V}s \nonumber \\&= -2 \; s^T\textbf{V}{{\,\mathrm{\textsf{d}}\,}}\textbf{G}a \nonumber \\&\quad + s^T\textbf{V}\textbf{G}\left( \textbf{G}^T\textbf{V}\textbf{G}\right) ^{-1} \left[ \left( {{\,\mathrm{\textsf{d}}\,}}\textbf{G}^T\textbf{V}\textbf{G}\right) + \left( \textbf{G}^T\textbf{V}{{\,\mathrm{\textsf{d}}\,}}\textbf{G}\right) \right] \left( \textbf{G}^T\textbf{V}\textbf{G}\right) ^{-1}\textbf{G}^T\textbf{V}s \nonumber \\&= -2 \; s^T\textbf{V}{{\,\mathrm{\textsf{d}}\,}}\textbf{G}a\nonumber \\&\quad + a^T \left[ \left( {{\,\mathrm{\textsf{d}}\,}}\textbf{G}^T\textbf{V}\textbf{G}\right) + \left( \textbf{G}^T\textbf{V}{{\,\mathrm{\textsf{d}}\,}}\textbf{G}\right) \right] a \nonumber \\&= -2 \; s^T\textbf{V}{{\,\mathrm{\textsf{d}}\,}}\textbf{G}a\nonumber \\&\quad +2 \; a^T \textbf{G}^T\textbf{V}{{\,\mathrm{\textsf{d}}\,}}\textbf{G}a \nonumber \\&= -2 \; \left( a^T \otimes s^T\textbf{V}\right) {{\,\mathrm{\textsf{d}}\,}}{{\,\textrm{vec}\,}}\textbf{G}\nonumber \\&\quad +2 \; \left( a^T \otimes a^T \textbf{G}^T \textbf{V}\right) {{\,\mathrm{\textsf{d}}\,}}{{\,\textrm{vec}\,}}\textbf{G}\nonumber \\&= -2 \; {{\,\textrm{vec}\,}}\left( \textbf{V}s a^T \right) ^T {{\,\mathrm{\textsf{d}}\,}}{{\,\textrm{vec}\,}}\textbf{G}\nonumber \\&\quad +2 \; {{\,\textrm{vec}\,}}\left( \textbf{V}\textbf{G}a a^T \right) ^T {{\,\mathrm{\textsf{d}}\,}}{{\,\textrm{vec}\,}}\textbf{G}\nonumber \\&= 2 \; {{\,\textrm{vec}\,}}\left( \textbf{V}\textbf{G}a a^T - \textbf{V}s a^T \right) ^T {{\,\mathrm{\textsf{d}}\,}}{{\,\textrm{vec}\,}}\textbf{G}\nonumber \\&= 2 \; {{\,\textrm{vec}\,}}\left( \left( \textbf{V}\textbf{G}a - \textbf{V}s\right) a^T\right) ^T {{\,\mathrm{\textsf{d}}\,}}{{\,\textrm{vec}\,}}\textbf{G}\nonumber \\&= \underbrace{2 \; {{\,\textrm{vec}\,}}\left( \left( \textbf{V}\textbf{G}a - \textbf{V}s\right) a^T\right) ^T {{\,\mathrm{\textsf{D}}\,}}\textbf{G}}_{= {{\,\mathrm{\textsf{D}}\,}}F_\text {SNLLS}} {{\,\mathrm{\textsf{d}}\,}}\theta _{\varvec{\Lambda }} \end{aligned}$$

## Appendix D: Alternative Proof

This is the analogous formulation to the one given in Kreiberg et al. (2021) for CFA models. We see that the resulting expressions contain very large Kronecker products; for reasons of computational efficiency, we therefore favor the expressions given in the main text. Let $\textbf{D}^+$ denote the Moore–Penrose inverse of the duplication matrix $\textbf{D}_{m_\textrm{obs}}$ from Magnus and Neudecker (2019b) such that

D1 $$\begin{aligned} \sigma = \textbf{D}^+{{\,\textrm{vec}\,}}(\varvec{\Sigma }) \end{aligned}$$

and $\textbf{L}$ be a matrix such that

D2 $$\begin{aligned} {{\,\textrm{vec}\,}}(\varvec{\Omega }) = \textbf{L}\; \theta _{\varvec{\Omega }} \end{aligned}$$

We can obtain $\textbf{L}\in \mathbb {R}^{m^2 \times \dim (\theta _{\varvec{\Omega }})}$ as

D3 $$\begin{aligned} \textbf{L}_{ij} = {\left\{ \begin{array}{ll} 1, &{} \text {if } i = (k-1)m + l \vee i = (l-1)m + k \text { with } (k, l) = {\mathcal {C}}_j\\ 0, &{} \text {otherwise} \end{array}\right. } \end{aligned}$$

and derive $\textbf{G}(\theta _{\varvec{\Lambda }})$ as

D4 $$\begin{aligned} \sigma (\theta )&= \textbf{D}^+ {{\,\textrm{vec}\,}}(\varvec{\Sigma }) \end{aligned}$$

D5 $$\begin{aligned}&= \textbf{D}^+ {{\,\textrm{vec}\,}}(\textbf{F}(\textbf{I}-\varvec{\Lambda })^{-1}\varvec{\Omega }(\textbf{I}-\varvec{\Lambda })^{-T}\textbf{F}^T) \end{aligned}$$

D6 $$\begin{aligned}&= \textbf{D}^+ \left( \textbf{F}\left( \textbf{I}-\varvec{\Lambda }\right) ^{-1}\otimes \textbf{F}\left( \textbf{I}-\varvec{\Lambda }\right) ^{-1}\right) {{\,\textrm{vec}\,}}(\varvec{\Omega }) \end{aligned}$$

D7 $$\begin{aligned}&= \underbrace{\textbf{D}^+ \left( \textbf{F}\left( \textbf{I}-\varvec{\Lambda }\right) ^{-1}\otimes \textbf{F}\left( \textbf{I}-\varvec{\Lambda }\right) ^{-1}\right) \textbf{L}}_{= G(\theta _{\varvec{\Lambda }})} \; \theta _{\varvec{\Omega }} \end{aligned}$$

We further define $\textbf{P}:= \left( \textbf{L}^T \otimes \textbf{D}^+ \left( \textbf{F}\otimes \textbf{F}\right) \right)$ and $\textbf{Q}:= \left( \textbf{I}_m \otimes \textbf{K}_m \otimes \textbf{I}_m \right)$, where ${\textbf {K}}_m$ is the commutation matrix from Magnus and Neudecker (2019b), and derive ${{\,\mathrm{\textsf{D}}\,}}\textbf{G}$ as

D8 $$\begin{aligned} {{\,\mathrm{\textsf{d}}\,}}{{\,\textrm{vec}\,}}\textbf{G}&= {{\,\mathrm{\textsf{d}}\,}}{{\,\textrm{vec}\,}}\left[ \textbf{D}^+ \left( \textbf{F}\left( \textbf{I}-\varvec{\Lambda }\right) ^{-1}\otimes \textbf{F}\left( \textbf{I}-\varvec{\Lambda }\right) ^{-1}\right) \textbf{L}\right] \nonumber \\&= {{\,\mathrm{\textsf{d}}\,}}{{\,\textrm{vec}\,}}\left[ \textbf{D}^+ \left( \textbf{F}\otimes \textbf{F}\right) \left( \left( \textbf{I}-\varvec{\Lambda }\right) ^{-1}\otimes \left( \textbf{I}-\varvec{\Lambda }\right) ^{-1}\right) \textbf{L}\right] \nonumber \\&= \left( \textbf{L}^T \otimes \textbf{D}^+ \left( \textbf{F}\otimes \textbf{F}\right) \right) {{\,\mathrm{\textsf{d}}\,}}{{\,\textrm{vec}\,}}\left( \left( \textbf{I}-\varvec{\Lambda }\right) ^{-1}\otimes \left( \textbf{I}-\varvec{\Lambda }\right) ^{-1}\right) \nonumber \\&= \textbf{P}{{\,\textrm{vec}\,}}\left( {{\,\mathrm{\textsf{d}}\,}}\left( \textbf{I}-\varvec{\Lambda }\right) ^{-1}\otimes \left( \textbf{I}-\varvec{\Lambda }\right) ^{-1} + \left( \textbf{I}-\varvec{\Lambda }\right) ^{-1}\otimes {{\,\mathrm{\textsf{d}}\,}}\left( \textbf{I}-\varvec{\Lambda }\right) ^{-1}\right) \nonumber \\&= \textbf{P}[ \nonumber \\&\quad {{\,\textrm{vec}\,}}\left( \left( \textbf{I}-\varvec{\Lambda }\right) ^{-1} {{\,\mathrm{\textsf{d}}\,}}\varvec{\Lambda }\left( \textbf{I}-\varvec{\Lambda }\right) ^{-1}\otimes \left( \textbf{I}-\varvec{\Lambda }\right) ^{-1}\right) \nonumber \\&\quad + {{\,\textrm{vec}\,}}\left( \left( \textbf{I}-\varvec{\Lambda }\right) ^{-1}\otimes \left( \textbf{I}-\varvec{\Lambda }\right) ^{-1} {{\,\mathrm{\textsf{d}}\,}}\varvec{\Lambda }\left( \textbf{I}-\varvec{\Lambda }\right) ^{-1}\right) \nonumber \\&\quad ] \nonumber \\&= \textbf{P}\textbf{Q}[ \nonumber \\&\quad {{\,\textrm{vec}\,}}\left( \left( \textbf{I}-\varvec{\Lambda }\right) ^{-1} {{\,\mathrm{\textsf{d}}\,}}\varvec{\Lambda }\left( \textbf{I}-\varvec{\Lambda }\right) ^{-1} \right) \otimes {{\,\textrm{vec}\,}}\left( \textbf{I}-\varvec{\Lambda }\right) ^{-1} \nonumber \\&\quad + {{\,\textrm{vec}\,}}\left( \textbf{I}-\varvec{\Lambda }\right) ^{-1} \otimes {{\,\textrm{vec}\,}}\left( \left( \textbf{I}-\varvec{\Lambda }\right) ^{-1} {{\,\mathrm{\textsf{d}}\,}}\varvec{\Lambda }\left( \textbf{I}-\varvec{\Lambda }\right) ^{-1}\right) \nonumber \\&\quad ] \nonumber \\&= \textbf{P}\textbf{Q}[ \nonumber \\&\quad \left( \textbf{I}_{m^2} \otimes {{\,\textrm{vec}\,}}\left( \textbf{I}-\varvec{\Lambda }\right) ^{-1} \right) {{\,\textrm{vec}\,}}\left( \left( \textbf{I}-\varvec{\Lambda }\right) ^{-1} {{\,\mathrm{\textsf{d}}\,}}\varvec{\Lambda }\left( \textbf{I}-\varvec{\Lambda }\right) ^{-1} \right) \nonumber \\&\quad + \left( {{\,\textrm{vec}\,}}\left( \textbf{I}-\varvec{\Lambda }\right) ^{-1} \otimes \textbf{I}_{m^2} \right) {{\,\textrm{vec}\,}}\left( \left( \textbf{I}-\varvec{\Lambda }\right) ^{-1} {{\,\mathrm{\textsf{d}}\,}}\varvec{\Lambda }\left( \textbf{I}-\varvec{\Lambda }\right) ^{-1} \right) \nonumber \\&\quad ] \nonumber \\&= \underbrace{\textbf{P}\textbf{Q}\left[ \left( \textbf{I}_{m^2} \otimes {{\,\textrm{vec}\,}}\left( \textbf{I}-\varvec{\Lambda }\right) ^{-1} \right) + \left( {{\,\textrm{vec}\,}}\left( \textbf{I}-\varvec{\Lambda }\right) ^{-1} \otimes \textbf{I}_{m^2} \right) \right] \left( \left( \textbf{I}-\varvec{\Lambda }\right) ^{-T} \otimes \left( \textbf{I}-\varvec{\Lambda }\right) ^{-1} \right) \textsf{D} \varvec{\Lambda }}_{= {{\,\mathrm{\textsf{D}}\,}}\textbf{G}} {{\,\mathrm{\textsf{d}}\,}}\theta _\Lambda \end{aligned}$$
